# Book Review: Cannabinoids and Neuropsychiatric Disorders

**DOI:** 10.3389/fpsyg.2021.697434

**Published:** 2021-05-28

**Authors:** Anthony L. Murkar, Rébecca Robillard

**Affiliations:** ^1^Sleep Research Unit, The Royal's Institute of Mental Health Research, Ottawa, ON, Canada; ^2^University of Ottawa School of Psychology, Ottawa, ON, Canada

**Keywords:** cannabis (marijuana), psychiatry, psychopathology, psychopharmacology, cannabinoids (THC)

## Introduction

Despite being quite well-studied, cannabis may be one of the most poorly understood compounds in molecular neuropsychiatry and pharmacology. This is partially due to the diverse behavioral effects of its constituent compounds themselves, the non-selective nature of the exogenous cannabinoids, and the legal hurdles affecting research outputs. The widespread use of marijuana for medical purposes (a complex mixture of over 400 chemical constituents found in widely varying quantities) rather than pure, isolated compounds also complicates the dissection of potential therapeutic applications. The book “*cannabinoids and neuropsychiatric disorders”* (Murillo-Rodriguez et al., 2021) provides an in-depth exploration of cannabis, its constituent molecules, and synthetic variants—as well as their effects on various psychiatric conditions. It provides the reader with a comprehensive introduction to the complex composition of *C. sativa* L. and our current understanding of the pharmacological actions of its constituent compounds (as well as the endogenous cannabinoids and synthetic products). It is a useful read for anyone with an interest in the psychopharmacology of medical cannabis, researchers, and healthcare practitioners alike.

## Summary

In the foreword to the book provided by R. Mechoulam, a critical problem of modern medicine's current stance on cannabinoids is highlighted: “medical cannabis,” a complex mixture of many compounds (often in unknown quantities, and many of whose actions we do not fully understand), is reaching the public as a poorly understood yet widely available medicinal product. In order for medical cannabis to be appropriately incorporated into clinical practice, it is therefore key for healthcare practitioners to have sufficient grasp of what they are prescribing and how it can affect the various conditions encountered in their practice. This book provides a timely synthesis, with an in-depth characterization of the different classes of cannabinoids and their activities.

Overall, the book consists of nine chapters providing the reader with a global overview of the current state of our pharmacological understanding of cannabinoids and the relevance of these compounds to various psychiatric conditions for which cannabis has been prescribed or studied. Chapter 1 is dedicated to the different varieties of cannabinoids while also covering many of the other non-cannabinoid compounds identified in the cannabis plant (e.g., the terpenoids) that could also possess their own therapeutic qualities. Chapters 2 and 3 then discuss the mechanisms believed to be responsible for the actions of cannabinoids. The first three chapters together provide an excellent overview of the cannabinoids and their activities. They also touch on the important and relatively recent discovery that many cannabinoids (both synthetic agonists and antagonists, as well as phytocannabinoids) can also act at novel orphan/recently deorphanized receptors (Begg et al., 2005; Ryberg et al., 2007; Huang et al., 2011; Brown et al., 2017) whose central distributions and functions we do not yet fully understand. Chapter 4 then discusses the clinical and pre-clinical evidence about the efficacy of cannabinoids garnered from clinical experimental models of both neuropsychiatric and non-neuropsychiatric conditions (with a particular emphasis on cancer, metabolic disorders/obesity, epilepsy, and neurodegenerative diseases).

The remaining chapters of the book then focus on the relevance of cannabinoids as interventions for various neuropsychiatric conditions. Chapters 5 through 8 contain an in-depth review of disorders for which cannabis has been the most well-studied—including depression [as well as its association with cannabis use disorder (CUD); Ch. 5], Alzheimer's disease (Ch. 6), Huntington's disease (Ch. 6), Parkinson's disease (Ch. 6), epilepsy (Ch. 7), and multiple sclerosis (Ch. 8). Finally, Chapter 9 concludes with a more general review of several additional disorders including anxiety disorders, schizophrenia, autism-spectrum disorders, attention-deficit hyperactivity disorder, and addiction (both in terms of CUD itself and of endocannabinoid-mediated modulation of the reward networks by other addictive substances). It also places a particular emphasis on what is known about modulation of the cannabinoid receptors in each condition.

## Evaluation

Overall, the book is clear, well-written, and provides an in-depth examination of the pharmacology of the cannabinoids that could be highly relevant for clinicians aiming to better understand products that their patients may be taking (either by prescription or outside of the guidance of healthcare practitioners) and the effects these products may have on neuropsychiatric conditions. It is also highly relevant for researchers wishing to develop a better understanding of the chemical characterization of these compounds and their effects on behavior.

We do find one important limitation with the book, however. Although it provides in-depth coverage of several psychiatric conditions, a chapter on post-traumatic stress disorder (PTSD) and/or other Trauma- and Stressor-Related Disorders is notably absent. This is despite that cannabinoids have been used quite extensively in the treatment of PTSD (Jetly et al., 2015; Steenkamp et al., 2017; Veterans Affairs Canada, 2019). Among conditions treated by cannabis, PTSD is perhaps the one with the strongest theoretical basis to support its indication for the condition (Lisboa et al., 2010; Neumeister, 2013; Neumeister et al., 2013; Pietrzak et al., 2014). High rates of co-occurring CUD with PTSD have also indicated this as a key area of concern with regards to mental health comorbidities (Bryan et al., 2021). Thus, it is one of the primary conditions associated with cannabis use that clinicians will likely seek to better understand.

## Discussion

Overall, the editors and authors of this book have presented an excellent resource for readers interested in the endocannabinoid system and its role in neuropsychiatry. Its coverage of the complex cannabinoid pharmacology, the actions of the cannabinoid receptors, and its thorough review of the constituents of the cannabis plant and their effects on various psychiatric conditions make it a helpful reference. Given the current landscape of cannabis use and emerging potential therapeutic applications, ascertaining a greater understanding of the intricacies of the cannabinoids is an area of great importance, both for research and clinical practice—and this book offers a great summary of the current state of the literature.

## Author Contributions

All authors listed made substantial contributions to the conception, formulation, writing, editing of this review, and read and approved the final manuscript.

## Conflict of Interest

The authors declare that the research was conducted in the absence of any commercial or financial relationships that could be construed as a potential conflict of interest.

## References

[B1] Begg M. Pacher P. Bátkai S. Osei-Hyiaman D. Offertáler L. Mo F. M.. (2005). Evidence for novel cannabinoid receptors. Pharmacol. Ther. 106, 133–145. 10.1016/j.pharmthera.2004.11.005 15866316

[B2] Brown K. J. Laun A. S. Song Z.-H. (2017). Cannabidiol, a novel inverse agonist for GPR12. Biochem. Biophys. Res. Commun. 493, 451–454. 10.1016/j.bbrc.2017.09.001 28888984 PMC5849771

[B3] Bryan J. L. Hogan J. Lindsay J. A. Ecker A. H. (2021). Cannabis use disorder and post-traumatic stress disorder: the prevalence of comorbidity in veterans of recent conflicts. J. Subst. Abuse Treat. 122:108254. 10.1016/j.jsat.2020.108254 33509412

[B4] Huang L. Ramirez J. C. Frampton G. A. Golden L. E. Quinn M. A. Pae H. Y.. (2011). Anandamide exerts its antiproliferative actions on cholangiocarcinoma by activation of the GPR55 receptor. Lab. Invest. 91, 1007–1017. 10.1038/labinvest.2011.62 21464819 PMC3126905

[B5] Jetly R. Heber A. Fraser G. Boisvert D. (2015). The efficacy of nabilone, a synthetic cannabinoid, in the treatment of PTSD-associated nightmares: a preliminary randomized, double-blind, placebo-controlled cross-over design study. Psychoneuroendocrinology 51, 585–588. 10.1016/j.psyneuen.2014.11.002 25467221

[B6] Lisboa S. F. Reis D. G. da Silva A. L. Corrêa F. M. Guimaraes F. S. Resstel L. B. (2010). Cannabinoid CB1 receptors in the medial prefrontal cortex modulate the expression of contextual fear conditioning. Int. J. Neuropsychopharmacol. 13, 1163–1173. 10.1017/S1461145710000684 20587131

[B7] Murillo-Rodriguez E. Pandi-Perumal S. R. Monti J. M. (2021). Cannabinoids and Neuropsychiatric Disorders. Cham: Springer. 10.1007/978-3-030-57369-0

[B8] Neumeister A. (2013). The endocannabinoid system provides an avenue for evidence-based treatment development for PTSD. Depress. Anxiety 30, 93–96. 10.1002/da.22031 23225490

[B9] Neumeister A. Normandin M. D. Pietrzak R. H. Piomelli D. Zheng M.-Q. Gujarro-Anton A.. (2013). Elevated brain cannabinoid CB1 receptor availability in post-traumatic stress disorder: a positron emission tomography study. Mol. Psychiatry 18, 1034–1040. 10.1038/mp.2013.61 23670490 PMC3752332

[B10] Pietrzak R. H. Huang Y. Corsi-Travali S. Zheng M.-Q. Lin S. Henry S.. (2014). Cannabinoid type 1 receptor availability in the amygdala mediates threat processing in trauma survivors. Neuropsychopharmacology 39:2519. 10.1038/npp.2014.110 24820537 PMC4207337

[B11] Ryberg E. Larsson N. Sjögren S. Hjorth S. Hermansson N.-O. Leonova J.. (2007). The orphan receptor GPR55 is a novel cannabinoid receptor. Br. J. Pharmacol. 152, 1092–1101. 10.1038/sj.bjp.0707460 17876302 PMC2095107

[B12] Steenkamp M. M. Blessing E. M. Galatzer-Levy I. R. Hollahan L. C. Anderson W. T. (2017). Marijuana and other cannabinoids as a treatment for posttraumatic stress disorder: a literature review. Depress. Anxiety 34, 207–216. 10.1002/da.22596 28245077

[B13] Veterans Affairs Canada (2019). Review of Marijuana for Medical Purposes - Veterans Affairs Canada. Available online at: https://www.veterans.gc.ca/eng/about-vac/publications-reports/reports/departmental-audit-evaluation/2016-review-marijuana-medical-purposes (accessed November 17, 2020).

